# Association between Livestock Ownership and Malaria Incidence in South-Central Ethiopia: A Cohort Study

**DOI:** 10.4269/ajtmh.22-0719

**Published:** 2023-04-24

**Authors:** Eskindir Loha

**Affiliations:** Centre for International Health, University of Bergen, Bergen, Norway;; Chr. Michelsen Institute, Bergen, Norway;; School of Public Health, Hawassa University, Hawassa, Ethiopia

## Abstract

Zooprophylaxis is one of the possible environmental vector control strategies for malaria prevention. However, its effect on reducing malaria transmission has been questionable, requiring a detailed understanding of contextual factors. This study aims to evaluate the effect of keeping livestock on malaria incidence in south-central Ethiopia. A cohort of 34,548 people in a total of 6,071 households was followed for 121 weeks from October 2014 to January 2017. Baseline data were collected, including livestock ownership. Weekly home visits were done to actively search for malaria cases, and passive case detection was also carried out. Malaria was diagnosed with rapid diagnostic tests. Log binomial and parametric regression survival-time models were used to estimate effect measures. A total of 27,471 residents had complete follow-ups, and the majority (87.5%) lived in households owning livestock, including cattle, sheep, goats, and chickens. The overall incidence risk of malaria was 3.7%, and there was a 24% reduction in the risk of malaria among livestock owners. The total cohort contributed to 71,861.62 person-years of observation. The incidence rate of malaria was 14.7 cases per 1,000 person-years. There was a 17% reduction in the rate of malaria among livestock owners. Meanwhile, the protective effect of livestock ownership increased as the number of livestock or the livestock-to-human ratio increased. In conclusion, livestock owners had less malaria. In a setup where domestication of livestock is a common practice and the predominant malaria vector tends to feed more on livestock than humans, zooprophylaxis remains a promising strategy for malaria prevention.

## INTRODUCTION

Zooprophylaxis is the use of animals to divert biting mosquitoes away from humans to prevent malaria infection. It was one of the recommended environmental measures for malaria vector control many years ago. It was considered an effective strategy in areas where the vectors that transmit malaria prefer to feed on animals (zoophilic) rather than on humans (anthropophilic). In addition, it could be implemented in areas where domestication of cattle, sheep, goats, or other livestock is common practice.^1^

However, the effect of zooprophylaxis remains debatable.^2^^–^^8^ Some researchers are in favor of it, whereas others reported it not to be beneficial, implying the need to evaluate its effect context-wise.^9^ It was argued that the presence of animals avails a blood meal source for mosquitoes and in effect contributes to their longevity, and thus their potential infectivity could be prolonged—the condition referred to as zoopotentiation; this might neutralize (even surpass) the desired zooprophylactic effect.^10^^,^^11^ Accordingly, to counterbalance such an undesired effect, strategies were proposed to enhance zooprophylaxis by, for instance, treating the animals with insecticides or separating their dwellings from humans and by other vector control measures, including the use of long-lasting insecticidal nets (LLINs) and indoor residual spraying with insecticides (IRS).^12^^–^^14^

Quantifying and projecting the effect of zooprophylaxis has been difficult, and concluding whether zooprophylaxis is a helpful strategy or not just by changes in entomological parameters makes it more complicated.^15^ For example, a study documented that, among collected mosquitoes, none was found to have the malaria parasite, making entomological inoculation rate (EIR) calculations impossible,^16^ even though its parallel epidemiological study confirmed the presence of cases of malaria.^17^ Such discrepancies could arise from (but are not limited to) mosquito sampling variations that complicate extrapolation of the effect of zooprophylaxis measured on entomological parameters to its impact on the overall incidence of malaria among humans. Although measuring the effect of zooprophylaxis using entomological parameters remains the best strategy, whenever parameters like EIR cannot be obtained, investigating the effect on human infections may be an option.

In Ethiopia, with an altitude < 2,000 m, the malaria parasite prevalence is generally low, at 1.2% in areas considered malarious.^18^ However, the heterogeneous nature of the transmission leads to high disease rates of epidemic potential in specific foci. The country is embarking on elimination, but maintaining the optimal benefits of the existing vector control mechanisms remains challenging.^19^ For example, a recent large cluster-randomized control trial investigated if combining interventions (LLINs with IRS) has an added value to either LLINs or IRS implemented alone. The result showed no added value. Surprisingly, it also documented no effect of either the combined or single intervention compared with the group that received neither. Consequently, residual malaria transmission and the low malaria incidence in the study area were speculated as possible reasons for not observing any effect of these proven prevention tools,^17^ but these results merit further investigation.

A review of the literature has suggested that an essential factor to consider when using zooprophylaxis as a malaria prevention strategy is whether the vectors are zoophilic or exophilic.^9^^,^^13^ Interestingly, the principal malaria vector in the study area was *Anopheles arabiensis*, which is zoophilic,^14^^,^^20^ biting often outdoors (exophilic) and before bedtime (before people are protected by bed nets).^16^ The bovine blood index of mosquitos collected outdoors was high (68%).^21^ This may imply the potential role of zooprophylaxis in the study area; this requires investigation. In addition, the fact that Ethiopia has the largest livestock population in Africa (65, 40, 51, and 49 million cattle, sheep, goats, and chickens, respectively)^22^ may encourage assessing this huge potential for malaria epidemiology in the country.

Therefore, taking advantage of the rich entomological information generated from the study area showing the exophilic and zoophilic behavior of the vector, the presence of data on distance from a potential malaria vector breeding place, demographic and economic characteristics, and the allocation of study participants to different trial arms for a large cluster-randomized controlled trial,^16^^,^^17^^,^^20^^,^^23^ this study aims to assess the effects of livestock ownership on the incidence of malaria in a large cohort of residents followed for 2 years in the Adami Tullu district in Oromia Regional State, Ethiopia.

## MATERIALS AND METHODS

This is a secondary analysis of the effects of livestock ownership on the incidence of malaria using a dataset from a large cluster-randomized controlled trial conducted to study the effect of a combination of IRS and LLINs versus a single intervention conducted from October 2014 to January 2017. This analysis was conducted in a framework of prospective cohort study design. The study area is located in the Adami Tullu district of the East Shewa Zone of the Oromia region in Ethiopia—in the Great Rift Valley with an altitude ranging between 1,500 m and 2,300 m. The capital of the district, Batu town, has a latitude of 7°56′N and a longitude of 38°42′E. Lake Ziway and Bulbula river are the main water bodies in the area. Farming and livestock rearing are the main means of subsistence in the area. Background information was collected at the beginning of the study, and a weekly follow-up visit to each household (HH) was conducted for a total of 121 weeks. Detailed descriptions of the main study setup, the dataset, and the results of the trial are provided elsewhere.^17^^,^^23^^,^^24^

### Exposure variable.

The exposure of interest was livestock ownership. The number of livestock owned at the HH level was obtained at the beginning of the study. The livestock-to-human ratio was also calculated.

### Outcome variable.

The outcome variable was malaria case incidence (having had at least one episode of malaria infection) during the whole follow-up period. Cases of malaria were identified by active (weekly follow-up to each HH) and passive (self-report to nearby tailored health facilities) means among those having had a fever or history of fever in the previous 48 hours. Both *Plasmodium falciparum* and *Plasmodium vivax* infections were counted. Blood tests were performed using rapid diagnostic test kits (CareStart® Malaria Pf/Pv combo test; Access Bio, Inc., Somerset, NJ).

### Potential confounders.

The list of potential confounders was limited to the available data from the main trial.^17^ This included sex, age, educational status of the HH head, the intervention arm to which the HH was allocated, the location/*Kebele* (*Kebele* is the lowest administrative structure in Ethiopia) of the HH, the distance of the HH from potential malaria vector breeding place, and wealth index. The water bodies along the Bulbula river and lake Ziway were the sites for malaria vectors to breed. The Global Positioning System (GPS) location of each HH and malaria vector breeding place was taken with a handheld GPS device (Garmin GPSMAP60CSx, Garmin International Inc., Olathe, KS). Proximity analysis using ArcMap 10.3.1 software was carried out to calculate the distance (in km) between each HH and the nearest potential vector breeding place.^25^ To generate the wealth index, a principal component factor analysis was done. A total of 13 variables were used including the main material of the wall, main material of the roof, ownership of agricultural land, having a separate room for the kitchen, access to electricity, and household assets (television, radio, watch, mobile phone, table, chair, bed, and bicycle). Four factors were retained explaining 52% of the variance. Oblique Promax rotation was used. The scale reliability coefficient was 0.72. The generated wealth scores were grouped into quintiles.

### Statistical model.

A log-binomial regression model was fitted to estimate the RR. For this model, all members of the HH were included in the analysis if they completed the total follow-up period of the main study (121 weeks; *N* = 27,471). The impact of a potential confounding variable on the crude estimate of the exposure variable was evaluated. The percentage change from the crude was the basis for deciding which variable to retain in the final model, and a ≥ 10% change was considered as a cut-off. Risk difference and RR along with 95% CIs were used to report the effect of livestock ownership on malaria risk.

In addition, the analysis was extended to account for the total follow-up time contributed by each member of the household in the estimation of an effect. For this, a parametric regression survival-time model was fitted to the whole dataset involving 34,548 people from a total of 6,071 HHs, with a total of 71,861.62 person-years of observation. Consequently, incidence rate difference and incidence rate ratio (IRR) with 95% CI were calculated. The analysis was carried out using Stata version 16 (StataCorp, College Station, TX).

## RESULTS

A total of 27,471 residents had complete follow-ups. The majority (87.5%) of study participants lived in a HH that owned livestock. The types of livestock included cow/ox, donkey/horse/mule, goat, sheep, and chickens. The majority of HHs with livestock owned a cow/ox (92%), with a median (IQR) of 4 (2–6). The majority of the HHs owned more than one type of livestock, and nearly one-fourth owned two, three, or four types of livestock. Slightly more than half (54%) of the study livestock owners had nine or more livestock of any type (Table 1).

**Table 1:** Owned livestock types and counts, Adami Tullu district, south-central Ethiopia, 2014–2017 (*N* = 24,047; 87.5% of 27,471)

Types and counts of livestock	HH members		Median (IQR) number of livestock
	*n*	%	
Type of livestock			
Cow/ox	22,016	91.55	4 (2–6)
Donkey/horse/mule	12,230	50.86	2 (1–2)
Goat	14,960	62.21	3 (2–6)
Sheep	4,872	20.26	3 (2–5)
Chickens	12,838	53.39	5 (3–9)
Number of types of livestock in the HH			
1	4,216	17.53	3 (2–4)
2	5,785	24.06	
3	6,547	27.23	
4	5,776	24.02	
5	1,723	7.17	
Number of any type of livestock			
Below 9	11,094	46.13	8 (3–16)
9 and above	12,953	53.87	

HH = household; IQR = intraquartile range.

The distribution of livestock ownership was almost similar across categories of variables initially considered as potential confounders (including the study arms of the main trial) except the location/*Kebele* of the HH and the distance of the HH from the potential malaria vector breeding place, for which the proportion ranged from 75.2% to 95.6% and median distance of 1.19 km versus 1.81 km, respectively (Supplemental Table 1).

A total of 1,013 of 27,471 cases of malaria were documented. The incidence of malaria was almost equal across different categories of potential confounders. Like the exposure variable distribution, the only exceptions observed were for the location/*Kebele* of the HH and the distance of the HH from the potential malaria vector breeding place, whereby we see a varying proportion that ranged between 0.8% to 13.9% and median distance of 1.76 km versus 1.33 km, respectively (Supplemental Table 2).

Among the available variables investigated for potential confounding effect, all variables except the location of the HH (*Kebele* and distance from vector breeding place) showed very little or no effect on the exposure variable estimate. The percentage change resulting from the inclusion of the location of the HH with reference to the potential malaria vector breeding place or the *Kebele* was almost 10% for the risk ratio, and this is the case only for the distance variable for the rate ratio analysis (Table 2). Because both Kebele and distance from the vector breeding site indicate the physical location of the HH, to be precise, distance from the vector breeding site of each HH was considered to adjust for the effect of the livestock ownership on malaria risk or rate.

**Table 2:** Adjusted effect measures and percentage change from the crude, Adami Tullu district, south-central Ethiopia, 2014–2017

Outcome: malaria	Livestock	X						
		Sex	Age	Education of HH head	Intervention arm	Kebele	Distance from vector breeding site in km	Wealth index
RR								
Crude	0.696							
Adjusted: Livestock + X		0.696	0.696	0.702	0.699	0.765	0.764	0.706
Percentage change (absolute) from crude: (Crude-Adjusted/Crude) × 100		0.011	0.022	0.764	0.383	9.87	9.804	1.467
IRR								
Crude	0.757							
Adjusted: Livestock + X		0.757	0.757	0.761	0.759	0.807	0.829	0.768
Percentage change (absolute) from crude: (Crude-Adjusted/Crude) × 100		0.006	0.000	0.544	0.272	6.598	9.407	1.441

HH = household; IRR = incidence rate ratio.

### Risk of malaria.

The overall incidence risk of malaria infection in the community was small (3.7%; 1,013 cases per 27,471 exposed individuals). Meanwhile, the risk was smaller for livestock owners (3.5% versus 5%), giving the absolute risk difference of 1.5% (95% CI: 0.8–2.3). The relative measure showed a 23.6% reduced risk of developing malaria among livestock owners, with an adjusted RR of 0.764 (95% CI: 0.651–0.898) (Table 3). Meanwhile, in the effort to see the dose–response relationship, the effect of livestock ownership increased as the number of livestock owned increased; that is, the protective efficacy increased from 12.9% for one to eight to 34.6% for nine or more livestock of any type (Table 4). To investigate further the dose-response, the livestock-to-human ratio was calculated. The average household size was 6.8 (SD: 2.4) people, and the median (IQR) livestock-to-human ratio was 1.38 (0.71–2.44). Consequently, the variable “livestock-to-human ratio” was grouped into tertiles for ease of presentation, and each tertile was then compared with a category with no livestock. Table 5 shows a 24.9% and 37.9% risk reduction if there were an average of 1.4 and 3.6 livestock per human in the HH, respectively.

**Table 3:** Effect of livestock ownership on malaria risk, Adami Tullu district, south-central Ethiopia, 2014–2017

Variable	HH owns livestock		Total
	Yes	No	
Malaria cases	841	172	1,013
No malaria	23,206	3,252	26,458
Total	24,047	3,424	27,471
Risk (%)	3.5	5.0	3.7
	Point estimate		95% CI
Absolute risk difference (%)	1.5		0.8–2.3
RR	0.696		0.593–0.817
RR*	0.764		0.651–0.898

HH = household.

* Adjusted for the distance of the HH from the vector breeding place.

**Table 4:** Dose-response relationship between livestock ownership and malaria risk, Adami Tullu district, south-central Ethiopia, 2014–2017

Variable	HH owns livestock			Total
	None	1–8	≥ 9	
Malaria cases	172	465	376	1,013
No malaria	3,252	10,629	12,577	26,458
Total	3,424	11,094	12,953	27,471
Risk (%)	5.0	4.2	2.9	3.7
RR (95% CI)	Ref	0.834 (0.703–0.990)	0.578 (0.484–0.689)	–
RR (95% CI)*	Ref	0.871 (0.734–1.033)	0.654 (0.547–0.783)	–

HH = household.

* Adjusted for the distance of the HH from the vector breeding place.

**Table 5:** Dose-response relationship between livestock-to-human ratio and malaria risk, Adami Tullu district, south-central Ethiopia, 2014–2017

Variable	Livestock to human ratio			
	No livestock	1st tertile (mean: 0.5)	2nd tertile (mean: 1.4)	3rd tertile (mean: 3.6)
Malaria	172	338	268	235
No malaria	3,252	7,367	7,409	8,430
Risk (%)	5.02	4.39	3.49	2.71
RR (95% CI)	Ref	0.873 (0.73–1.045)	0.695 (0.576–0.838)	0.54 (0.445–0.655)
RR (95% CI)*	Ref	0.902 (0.754–1.079)	0.751 (0.622–0.906)	0.621 (0.51–0.755)

* Adjusted for the distance of the household from the vector breeding place.

### Rate of malaria.

For this analysis, a total sample size of 34,548 was used, and the total number of cases of malaria was 1,059. The parametric regression survival-time model revealed a reduction in the malaria rate by 17.1% for those who reported livestock ownership, with an adjusted IRR of 0.829 (95% CI: 0.704–0.975) (Table 6). There was a further reduction in the malaria rate (by 26.8%) among those owning nine or more livestock, with an adjusted IRR of 0.732 (95% CI: 0.611–0.877) (Table 7). As the livestock-to-human ratio increased, the malaria rate decreased by 19.2% (for an average of 1.4 livestock per human) and then by 29% (for an average of 3.6 livestock per human) (Table 8).

**Table 6:** Effect of livestock ownership on malaria rate, Adami Tullu district, south-central Ethiopia, 2014–2017

Variable	HH owns livestock		Total
	Yes	No	
Malaria cases	883	176	1,059
Person-years	62,436.94	9,424.67	71,861.62
Incidence rate (per 1,000 person-years)	14.1	18.7	14.7
Incidence rate difference (95% CI) (per 1,000 person-years)	4.5 (1.6–7.4)		
IRR (95% CI)	0.757 (0.643–0.895)		
IRR (95% CI)*	0.829 (0.704–0.975)		

HH = household; IRR = incidence rate ratio.

* Adjusted for the distance of the HH from the vector breeding place.

**Table 7:** Dose-response relationship between livestock ownership and malaria rate, Adami Tullu district, south-central Ethiopia, 2014–2017

Variable	HH owns livestock		
	None	1–8	≥ 9
Malaria	176	481	402
Person-years	9,424.67	29,215.9	33,221.04
Incidence rate (per 1000 person-years)	18.7	16.5	12.1
IRR (95% CI)	Ref	0.882 (0.742–1.048)	0.648 (0.543–0.774)
IRR (95% CI)*	Ref	0.92 (0.774–1.094)	0.732 (0.611–0.877)

HH = household; IRR = incidence rate ratio.

* Adjusted for the distance of the HH from the vector breeding place.

**Table 8:** Dose-response relationship between livestock to human ratio and malaria rate, Adami Tullu district, south-central Ethiopia, 2014–2017

Variable	Livestock to human ratio			
	No livestock	1st tertile (mean: 0.5)	2nd tertile (mean: 1.4)	3rd tertile (mean: 3.6)
Malaria	176	349	278	256
Person-years	9,424.67	20,373.33	19,888.87	22,174.75
Incidence rate (per 1,000 person-years)	18.7	17.13	13.98	11.55
IRR (95% CI)	Ref	0.917 (0.765–1.1)	0.748 (0.62–0.904)	0.618 (0.51–0.749)
IRR (95% CI)*	Ref	0.948 (0.791–1.136)	0.808 (0.668–0.976)	0.71 (0.584–0.864)

IRR = incidence rate ratio.

* Adjusted for the distance of the household from the vector breeding place.

## DISCUSSION

There was a 24% reduction in the risk of acquiring malaria infection if the HH owns livestock after adjusting for proximity of the household to vector breeding habitat. The protective effect increased to 34% for those having nine or more livestock. Similarly, there was a 17% reduction in the rate of malaria among the exposed. Living with many livestock showed a further reduction of the malaria rate by 27%. The absolute reductions (1.5% or 4.5 cases per 1,000 person-years) may seem negligible, but because the overall malaria risk (3.7%) and rate (14.7 cases per 1,000 person-years) in the study area are low, the absolute reductions observed could be considered proportionally sizable and of public health importance.

This study showed that the greater the number of livestock, the lower the risk of malaria. This may imply that an increased animal blood meal availability might have resulted in an effective diversion of malaria mosquitoes away from humans. The increase in the number of domestic animals may also accompany a risk of increasing the lifespan of the vector; however, if the vectors were attracted to animals and prefer to be fed outdoors, having livestock around may not always increase the malaria risk (zoopotentiation). A model by Sota and Mogi^5^ showed that to have the expected benefit of zooprophylaxis, there must be an increase in the number of domestic animals, given the malaria infection rate remained low. In line with this notion, it was interesting to see the relative abundance of livestock to the household size. The result was consistent in that the greater the livestock-to-human ratio was, the greater the protective effect.

In the main trial, both indoor malaria prevention tools (IRS with LLINs or each alone) did not show a difference in reducing malaria incidence compared with the control arm. The plausible reason provided was residual malaria transmission. Likewise, the entomological finding from the same study setup indicated that the principal malaria vector in the area demonstrated exophilic behavior and early biting (before bedtime), leading to speculation that an outdoor malaria transmission potential is more likely.^16^ In such a circumstance, we may consider livestock ownership to have played at least some role in malaria transmission dynamics, although the overall human blood index of *An. arabiensis* was higher compared with the bovine blood index (69% versus 39%) and the bovine blood index of those collected outdoors was higher than that indoors (68% versus 27%).^21^ This may indicate that livestock was around to feed the zoophilic mosquitoes outdoors in close vicinity of the owners. The pits that were used as outdoor mosquito resting shelters (and their subsequent collection spot) were also dug in a compound of the main house.^20^^,^^21^ In addition, the more abundant and exophilic vector species in the area, *An. zeimanni*,^16^ was reported to feed more on cattle than on humans, with a bovine and human blood index of 67% and 50%, respectively.^21^ Therefore, if residual confounding could not explain the observed effect of livestock ownership on the risk or rate of malaria infection, given the feeding preferences of the *An. arabiensis* (principal) and also *An. Zeimanni* (potential and more abundant) vectors of malaria in the locality, the common practice of domestication of animals (88%) in this location might have contributed to the suppression of human malaria infections to a greater extent.

Meanwhile, LLINs are among the approved malaria prevention tools. In the study area, the proportion of individuals sleeping under the net the night before the interview never surpassed 60%, even at the start of the study, despite universal coverage being achieved. The coverage of LLINs decreased from 100% to as low as 4% in 2 years; the goal was that the HHs would preserve the LLINs in a functional state for 3 years, but most of the distributed LLINs were either thrown away or used for unintended purposes.^26^^,^^27^ Therefore, in such a setup where we could not maintain optimal coverage and use of LLINs and where the predominant malaria vector has a zoophilic tendency, considering zooprophylaxis may be sensible. The proposed treatment of cattle with insecticides may need to be revitalized to maximize the benefit of zooprophylaxis.

This study has limitations. Most importantly, the exposure measurement was limited to the availability and count of livestock at the start of the study period, whereas malaria incidence was measured longitudinally. Because there were no data on whether the HH kept the livestock for the whole period of the study, the conclusions from this study should be taken with caution as the HH may not keep the livestock until the end of the follow-up period, and each member of the household may not get similar and sustained protection. In addition, the place to keep the livestock also matters for the effective diversion of mosquito bites, but there were no data on it. Therefore, it was impossible to report the effect of the distance of the animal quarters from or having the livestock inside or outside of the human dwelling. Nevertheless, with all such limitations, this study may shed light on the potential of this long-standing but poorly understood vector control mechanism in areas where the context favors it. Further research is warranted with better exposure measurement.

## Financial Disclosure

The main study was supported by the Research Council of Norway through the GLOBVAC program (Project Number: 220554). This secondary analysis was supported by the Centre for Intervention Science in Maternal and Child Health (CISMAC; Project number 223269), which is funded by the Research Council of Norway through its Centres of Excellence scheme and the University of Bergen, Norway.

## Supplemental Materials


Supplemental materials
