# Supplementary material for: Association between Livestock Ownership and Malaria Incidence in South-Central Ethiopia: A Cohort Study
Source: Am J Trop Med Hyg. 2023 Apr 24;108(6):1145–50. doi: 10.4269/ajtmh.22-0719 (PMC10540100; doi:10.4269/ajtmh.22-0719)
Supplement: Supplementary file 1 [file tpmd220719.SD1.pdf]

Supplementary Table 1. Cross tabulations of livestock ownership and potential confounders, Adami Tullu district, south-central Ethiopia, 2014–2017

n=27,471		HH owns a livestock			
		No (n=3,424)		Yes (n= 24,047)	
		No.	%	No.	%
Sex	Male	1,748	12.6	12,149	87.4
	Female	1,676	12.3	11,898	87.7
Age group	Under 5	637	14.9	3,650	85.1
	5 to 14	970	10.3	8,429	89.7
	15+	1,817	13.2	11,968	86.8
Education of HH head	Illiterate	1,828	11.5	14,039	88.5
	Read and write	395	13.5	2,540	86.5
	Formal education	1,201	13.9	7,468	86.1
Intervention arm to which the HH was assigned	IRS +ITN	946	13.8	5,914	86.2
	ITN	887	12.8	6,034	87.2
	IRS	751	12.2	5,402	87.8
	Routine	840	11.1	6,697	88.9
Location/*Kebele*	Walin Bula	473	24.8	1,432	75.2
	Negalegn	168	19.3	703	80.7
	Elka Chelemo	301	14.7	1,745	85.3
	Edo Gojola	167	6.6	2,374	93.4
	Abine Geremama	691	23.2	2,290	76.8
	Qamo Garbi	62	5.5	1,063	94.5
	Garbi Widena Boramo	106	7.4	1,321	92.6
	Halaku	67	11.2	531	88.8
	Anano Shisho	213	7.0	2,818	93.0
	Golba Aluto	168	11.7	1,269	88.3
	Dodicha	256	9.1	2,561	90.9
	Boccessa	556	24.4	1,726	75.6
	Abayi Deneba	196	4.4	4,214	95.6
Wealth index	Poor	736	14.48	4,348	85.52
	Second	793	13.71	4,989	86.29
	Middle	758	13.4	4,897	86.6
	Forth	500	9.6	4,710	90.4
	Rich	637	11.1	5,103	88.9
Distance from vector breeding site in km: Median (IQR)		1.19 (0.47-2.13)		1.81(0.83-3.12)	

HH: Household; *Kebele* is the lowest administrative structure in Ethiopia

Supplementary Table 2. Cross tabulations of malaria cases and potential confounders, Adami Tullu district, south-central Ethiopia, 2014–2017

n=27,471		Had malaria			
		No (n=26,458)		Yes (n=1,013)	
		No.	%	No.	%
Sex	Male	13,393	96.4	504	3.6
	Female	13,065	96.3	509	3.7
Age group	Under 5	4,047	94.4	240	5.6
	5 to 14	9,074	96.5	325	3.5
	15+	13,337	96.8	448	3.2
Education of HH head	Illiterate	15,324	96.6	543	3.4
	Read and write	2,793	95.2	142	4.8
	Formal education	8,341	96.2	328	3.8
Intervention arm to which the HH was assigned	IRS +ITN	6,589	96.0	271	4.0
	ITN	6,667	96.3	254	3.7
	IRS	5,922	96.2	231	3.8
	Routine	7,280	96.6	257	3.4
Location/*Kebele*	Walin Bula	1,820	95.5	85	4.5
	Negalegn	750	86.1	121	13.9
	Elka Chelemo	1,894	92.6	152	7.4
	Edo Gojola	2,500	98.4	41	1.6
	Abine Geremama	2,920	98.0	61	2.0
	Qamo Garbi	1,071	95.2	54	4.8
	Garbi Widena Boramo	1,371	96.1	56	3.9
	Halaku	589	98.5	9	1.5
	Anano Shisho	3,006	99.2	25	0.8
	Golba Aluto	1,392	96.9	45	3.1
	Dodicha	2,609	92.6	208	7.4
	Boccessa	2,212	96.9	70	3.1
	Abayi Deneba	4,324	98.0	86	2.0
Wealth index	Poor	4,868	95.75	216	4.25
	Second	5,543	95.87	239	4.13
	Middle	5,452	96.41	203	3.59
	Forth	5,035	96.64	175	3.36
	Rich	5,560	96.86	180	3.14
Distance from vector breeding site in km: Median (IQR)		1.76 (0.76-3.05)		1.33 (0.58-2.20)	

HH: Household; *Kebele* is the lowest administrative structure in Ethiopia
